# Awareness of Oral and Maxillofacial Surgery as a Career Choice Among Dental Students: An Online Questionnaire Survey

**DOI:** 10.7759/cureus.67039

**Published:** 2024-08-16

**Authors:** Munish Kumar, Akshit Vermani, Neeraj Attri, Mohit Verma, Faiyaz Alam, Rishabh Kasrija

**Affiliations:** 1 Department of Oral and Maxillofacial Surgery, Guru Nanak Dev Dental College and Research Institute, Sunam, IND; 2 Department of Oral and Maxillofacial Surgery, JSS Dental College, Mysuru, IND

**Keywords:** survey, oral surgery, dental students, career choice, awareness

## Abstract

Introduction: Most dental students are interested in pursuing a postgraduate degree after completing their Bachelor of Dental Surgery (BDS). Among the various specialties, oral surgery, which bridges the fields of medicine and dentistry, is the most attractive option for many young dental professionals. This study aimed to explore the factors influencing students' choices for postgraduation in oral and maxillofacial surgery (OMFS) and to assess their knowledge, attitudes, and perceptions (KAP) towards this branch of dentistry.

Materials and methods: An online survey was conducted with 450 third- and final-year BDS students and interns from various dental colleges. The survey included a self-administered questionnaire designed to collect demographic information and insights into students' motivations and KAP regarding postgraduation in OMFS. The questionnaire was distributed via an internet-based survey program and shared through WhatsApp Messenger accompanied by a consent form. To determine the significance between variables, non-parametric test, such as the chi-square test, was employed. The KAP scores were calculated and represented as mean ± standard deviation (SD), and intergroup comparisons were performed using the two-way independent T-test (for two groups) and two-way analysis of variance (ANOVA) test (for three groups). The level of significance was set at P ≤0.05.

Results: The sample comprised 320 (71%) females and 130 (29%) males. A total of 285 (63.33%) respondents wanted to pursue postgraduation after BDS, whereas 130 (28.89%) did not. Long working hours, risk and liability, and lack of skills were the main reasons for not opting for postgraduation in OMFS as a career option. The primary reasons cited for choosing OMFS included better career prospects (75.08%%) and the influence of the mentor (59.65%). The main benefits of choosing postgraduation in OMFS were advanced skills (88.77%), and high earning (85.61%). The mean KAP scores were higher for government institutions in urban areas and for males.

Conclusion: This study revealed that a substantial number of undergraduate dental students were inclined to specialize in OMFS after graduation. The combination of medicine and dentistry significantly influences the choice of OMFS as a career option.

## Introduction

Dentistry is widely regarded as a sought-after and financially rewarding vocation in numerous countries [1]. The selection of a profession often differs between societies due to a range of factors, including gender, ethnicity, familial and environmental attributes, as well as the educational achievements of parents [2]. A career can be described as an individual's metaphorical journey through learning, work, and other life aspects. Career options refer to the extent to which a person seeks involvement in a career that is central to their life. The expectation of a secure and bright professional future motivates students to choose their career paths.

For decades, medical and dental students, in particular, have developed a specific interest in one of the subjects during their course of study. Reasons for this can vary from person to person. They are often attracted to these courses because of their associated social and professional status, improved quality of life and income, recommendations from others, and the opportunity to play a role in community health services. The majority of these students aim to pursue a postgraduate degree after completing their graduation [3]. Oral and Maxillofacial Surgery (OMFS) is a specialized branch of dentistry that deals with the diagnosis and treatment of various pathologies, trauma, and deformities of the orofacial region [4]. The scope of OMFS ranges from simple tooth extraction to complex procedures, such as surgical extraction of impacted teeth, treatment of facial space infections, trauma care, facial clefts, orthognathic surgery, head and neck malignancy treatment, temporomandibular joint disorders, salivary gland diseases, pre-prosthetic surgery, implants, and various complex craniomaxillofacial procedures. This wide array of specialties necessitates training in both medicine and dentistry [5]. According to previous studies, most dental students lack awareness of this field and do not understand its scope [4-6]. However, all these studies were conducted in Western countries.

Therefore, due to the lack of data on the perception and awareness of dental students regarding OMFS, the present study was conducted to analyze the knowledge, attitude, and perception (KAP) of dental students in choosing OMFS as a career option for pursuing postgraduation after completion of an undergraduate course.

## Materials and methods

Study design and setting

The present cross-sectional, descriptive, questionnaire-based study was conducted from 1st March to 30th April 2024, by the Department of OMFS on undergraduate completed third- and final-year Bachelor of Dental Surgery (BDS) and interns from the dental colleges of North India, who were registered with the Dental Council of India (www.dciindia.gov.in). This survey was approved by the Institutional Ethical Committee of Guru Nanak Dev Dental College (GNDDC/IERB/04/2024/08) and conducted in accordance with the ethical principles outlined in the Declaration of Helsinki. The questionnaire was written based on the Checklist for Reporting Results of Internet E-Surveys (CHERRIES) [7]. The first section of the questionnaire's informed consent form was structured, including the study objectives, target population, principle of voluntariness, and other essential elements.

Sample size estimation

The sample size for this study was determined using the calculator net sample size calculator, based on a confidence level of 95% and a margin of error of 5%. The assumed population proportion of BDS students opting for postgraduate courses was 50% [8], with a total population of 6,900 students across 30 dental colleges in three states (Punjab, Haryana, and Himachal Pradesh), encompassing students in the third and final year of BDS, as well as those in internships. The minimum required sample size, calculated to ensure statistical reliability and representativeness, was 365. To ensure comprehensive representation, a stratified random sampling method was employed, with an equal number of respondents selected from each college, resulting in a final sample size of 450 respondents for the study. 

Respondents, data collection and pretesting

The roster comprising dental institutions was procured from the official website of the Dental Council of India, including their respective email contacts and telephone numbers. A formal correspondence endorsed by the departmental head was dispatched to the aforementioned dental colleges, soliciting the provision of a roster delineating third- and final-year undergraduate students and interns within the college, accompanied by their email contacts and telephone numbers. An online survey was administered via a questionnaire that was randomly distributed through an Internet-based survey program (Google Forms: [https://forms.gle/w8SGfvtkjPxR8nZx7]). An invitation to participate, including a consent form and link to the survey, was sent via WhatsApp Messenger. Respondents were given the opportunity to complete the questionnaire on a single occasion. The principal investigator was the sole individual who granted access to data. Duplicated entries were rectified, and only fully completed responses were considered. Reminder messages were sent after one month to complete the forms. Undergraduate third- and final-year students and interns who agreed to participate in the study were included. Respondents who did not provide their consent and those with incomplete forms were excluded from the study.

The questionnaire was divided into three domains. The first domain, known as Part A, focused on five open-ended questions on sociodemographic characteristics in which respondents entered their age, gender, year of study, workplace, and region. Part B consisted of six closed-ended questions, identifying the respondents’ knowledge about OMFS as a career option for pursuing postgraduation (four questions used a Likert three-point scale (yes, not sure, no), and two were multiple-choice questions). Part C consisted of five closed-ended questions identifying the respondents’ attitudes and perceptions (four questions used a Likert three-point scale, and one was a multiple-choice question). Each question (except for multiple choices) was scored as Yes=1, Not sure=0, or No=-1. The questionnaire was created through the collaborative efforts of five professionals, including three oral surgeons, one artificial intelligence (AI) expert, and one researcher expert with a decade of experience, who were not part of the study. Subsequent to the evaluation conducted by these five specialists, Aiken's V statistic was calculated to be 0.94, indicating a favourable level of content validity. To assess the dependability of the inquiries, a preliminary examination or pretesting of the questionnaire was conducted on 35 individuals who were not involved in the study. The reliability of the questionnaire was assessed using Cronbach's alpha, which yielded a value of 0.88. To determine the level of agreement among the questions, the questionnaire was administered to the same cohort after a two-week interval. Inter-observer agreement was evaluated using kappa coefficient, which yielded a value of 0.94.

Statistical analysis

The collected data were subjected to statistical analysis using the SPSS software version 22 (IBM Corp., Armonk, NY, USA). The normal distribution of the data was examined using the Shapiro-Wilk test. Compilation and presentation of sociodemographic variables and respondents' responses were achieved using a frequency distribution in the form of n% and parametric data in the form of mean ± standard deviation (SD). To determine the significance between variables, a non-parametric test, such as the chi-square test, was employed. The KAP scores were calculated and represented as mean ± SD and intergroup comparisons were performed using the two-way independent T-test (for two groups) and two-way analysis of variance (ANOVA) test (for three groups). The level of significance was set at P ≤0.05.

## Results

Three hundred twenty (71%) female respondents responded compared to 130 (28.88%) male respondents in the age range of 20-28 years. A total of 118 (26.22%) females were in the third-year BDS, whereas 52 (11.56%) were in the final year of BDS. A total of 390 (86.67%) respondents belonged to private institutions and 240 (53.33%) belonged to rural areas (Table 1).

**Table 1:** Demographic details of the participants BDS: Bachelor of Dental Surgery; Data represented in form of n (%)

Variables	Male n (%)	Female n (%)	Total n (%)
	130 (28.88)	320 (71.12)	450 (100.00)
Age group (years)			
20-22	78 (17.33)	167 (37.11)	245 (54.44)
23-25	36 (8.00)	134 (29.78)	170 (37.78)
26-28	16 (3.56)	19 (4.22)	35 (7.78)
Type of institution			
Private	112 (24.89)	278 (61.78)	390 (86.67)
Government	18 (4.00)	42 (9.33)	60 (13.33)
Location of institution			
Rural	84 (18.67)	156 (34.67)	240 (53.33)
Urban	46 (10.22)	164 (36.44)	210 (47.67)
Level of study			
Intern	46 (10.22)	104 (23.11)	150 (33.33)
4th BDS	52 (11.56)	98 (21.78)	150 (33.33)
3rd BDS	32 (7.11)	118 (26.22)	150 (33.33)

A total of 285 (63.33%) respondents wanted to pursue postgraduation after BDS, whereas 130 (28.89%) did not. Long working hours, risk and liability, and lack of skills were the main reasons for not opting for postgraduation in OMFS as a career option. The influence of the mentor was the main reason for choosing postgraduation in OMFS, with a statistically significant difference (p=.001). A total of 155 (54.39%) respondents were aware of the different treatment modalities in OMFS with statistically significant difference (p=.013). A total of 164 (57.54%) respondents felt that clinical exposure to BDS influenced their decision to consider OMFS for postgraduation. A total of 170 (59.65%) respondents felt that their institution was very supportive of providing proper career guidance to students. A total of 194 (68.07%) respondents felt that OMFS had a great future, and 171 (60.00%) felt that OMFS was more competitive than any other dental specialty. The main benefits of pursuing postgraduate programs in OMFS are high earnings, reputation, and advanced skills. Of the respondents, 244 (85.61%) felt that postgraduation in OMFS had financial benefits, and 200 (70.18%) felt that their BDS grades would influence their choice of specialization (Table 2).

**Table 2:** Comparison of responses between levels of study of participants *p<0.05: Significant; BDS: Bachelor of Dental Surgery; OMFS: Oral and Maxillofacial Surgery; Data represented in form of n (%)

Knowledge	Response	Intern, n(%) 150 (33.33)	4th BDS, n(%) 150 (33.33)	3rd BDS, n(%) 150 (33.33)	Total, n(%)	Chi-square value
Are you interested in pursuing a postgraduate course in OMFS after BDS?	Yes	97 (64.67)	90 (60.00)	98 (65.33)	285 (63.33)	0.747
	No	43 (28.67)	45 (30.00)	42 (28.00)	130 (28.89)	
	Not sure	10 (6.67)	15 (10.00)	10 (6.67)	35 (7.78)	
If no, what are the barriers in choosing the OMFS as career option? (n=130)	Long working hours	42 (32.31)	48 (36.92)	52 (40.00)	142 (31.56)	0.642
	Risk and liability	42 (32.31)	43 (33.08)	52 (40.00)	137 (30.44)	
	Blood exposure	5 (3.85)	12 (9.23)	22 (16.92)	39 (8.67)	
	Lack of skills	35 (26.92)	42 (32.31)	45 (34.62)	122 (27.11)	
	Other branch	30 (23.08)	35 (26.92)	45 (34.62)	110 (24.44)	
	No postgraduation	15 (11.54)	20 (15.38)	20 (15.38)	55 (12.22)	
If yes, what are the main reasons for choosing this specialization? (n=285)	Personal choice	30 (10.53)	49 (17.19)	41 (14.39)	120 (26.67)	0.001*
	Mentor influence	50 (17.54)	62 (21.75)	58 (20.35)	170 (37.78)	
	Financial stability	74 (25.96)	36 (12.63)	24 (8.42)	134 (29.78)	
	Career prospects	76 (26.67)	76 (26.67)	62 (21.75)	214 (47.56)	
Are you aware of different treatment modalities in OMFS?	Yes	65 (61.90)	55 (57.89)	35 (36.84)	155 (54.39)	0.013*
	No	25 (23.81)	15 (15.79)	28 (29.47)	68 (23.86)	
	Not sure	15 (14.29)	20 (21.05)	22 (23.16)	57 (20.00)	
How has your clinical exposure to OMFS during BDS influenced your decision to consider this specialization?	Significant	65 (61.90)	55 (57.89)	44 (46.32)	164 (57.54)	0.177
	Moderate	24 (22.86)	15 (15.79)	16 (16.84)	55 (19.30)	
	Not significant	16 (15.24)	20 (21.05)	25 (26.32)	61 (21.40)	
How supportive do you find your institution and faculty in guiding students toward postgraduate studies?	Very supportive	77 (73.33)	48 (50.53)	45 (47.37)	170 (59.65)	0.008*
	Moderate	20 (19.05)	32 (33.68)	30 (31.58)	82 (28.77)	
	Not supportive	8 (7.62)	15 (15.79)	10 (10.53)	33 (11.58)	
Attitude and perception						
Do you think OMFS has great future?	Yes	72 (68.57)	67 (70.53)	55 (57.89)	194 (68.07)	0.46
	No	20 (19.05)	13 (13.68)	12 (12.63)	45 (15.79)	
	Not sure	13 (12.38)	15 (15.79)	18 (18.95)	46 (16.14)	
Do you think oral surgery as a career is more competitive than any other speciality?	Yes	70 (66.67)	56 (58.95)	45 (47.37)	171 (60.00)	0.122
	No	25 (23.81)	23 (24.21)	20 (21.05)	68 (23.86)	
	Not sure	10 (9.52)	16 (16.84)	20 (21.05)	46 (16.14)	
What do you perceive as the main benefits of specializing in OMFS?	High earning	90 (31.58)	82 (28.77)	72 (25.26)	244 (85.61)	0.99
	Reputation	85 (29.82)	79 (27.72)	68 (23.86)	232 (81.40)	
	Advanced skills	95 (33.33)	80 (28.07)	78 (27.37)	253 (88.77)	
	Personal choice	65 (22.81)	54 (18.95)	56 (19.65)	175 (61.40)	
How significant are the financial considerations in your decision to pursue postgraduate studies in OMFS?	Significant	50 (47.62)	38 (36.19)	32 (30.48)	120 (42.11)	0.39
	Moderate	37 (35.24)	45 (42.86)	38 (36.19)	120 (42.11)	
	Not significant	18 (17.14)	12 (11.43)	15 (14.29)	45 (15.79)	
Do you believe your BDS grades influence your choice of postgraduate specialization?	Yes	78 (74.29)	72 (68.57)	50 (47.62)	200 (70.18)	0.09
	No	20 (19.05)	15 (14.29)	25 (23.81)	60 (21.05)	
	Not sure	7 (6.67)	8 (7.62)	10 (9.52)	25 (8.77)	

When reasons for not opting for postgraduation in OMFS were evaluated, 110 (84.61%) respondents gave the reason for long working hours, while 85 (65.38%) respondents chose other specializations. Most respondents (78.46%) belonged to private institutions. The main reasons for interns and third and final-year BDS students were long working hours and the associated risks and liabilities. A total of 105 (80.77%) respondents in the rural sector and 31 (23.84%) did not want to pursue post-graduation after BDS (Table 3).

**Table 3:** Participants' distribution according to reason for not opting for OMFS as postgraduation (PG) course BDS: Bachelor of Dental Surgery; OMFS: Oral and maxillofacial Surgery; Data represented in form of n (%)

Reasons not to choose OMFS	n (%)	Long working hours, n (%)	Risk and liability, n (%)	Blood exposure, n (%)	Lack of skills, n (%)	Other branches, n (%)	No PG, n (%)
Total responses	130 (29)	110 (84.61)	95 (73.07)	40 (30.76)	110 (84.61)	85 (65.38)	31 (23.84)
Type of institution							
Private	102 (78.46)	95 (73.08)	80 (61.54)	35 (26.92)	90 (69.23)	77 (59.23)	23 (17.69)
Government	28 (21.54)	15 (11.54)	15 (11.54)	5 (3.85)	20 (15.38)	8 (6.15)	8 (6.15)
Location of institution							
Rural	105 (80.77)	88 (67.69)	85 (65.38)	31 (23.85)	94 (72.31)	70 (53.85)	26 (20.00)
Urban	25 (19.23)	22 (16.92)	10 (7.69)	9 (6.92)	16 (12.31)	15 (11.54)	5 (3.85)
Level of study							
Intern	30 (23.08)	20 (15.38)	18 (13.85)	5 (3.85)	20 (15.38)	18 (13.85)	5 (3.85)
4th BDS	40 (30.77)	35 (26.92)	35 (26.92)	12 (9.23)	45 (34.62)	32 (24.62)	12 (9.23)
3rd BDS	60 (46.15)	55 (42.31)	42 (32.31)	23 (17.69)	45 (34.62)	35 (26.92)	14 (10.77)
Gender							
Male	35 (26.92)	25 (19.23)	10 (7.69)	8 (6.15)	25 (19.23)	20 (15.38)	11 (8.46)
Female	95 (73.08)	85 (65.38)	85 (65.38)	32 (24.62)	85 (65.38)	65 (50.00)	20 (15.38)

When reasons for opting for postgraduation in OMFS were evaluated, 214 (75.08%) respondents gave the reason for better career prospects, while 170 (59.65%) respondents gave the reason for mentor influence. Most respondents (86.67%) belonged to private institutions. A total of 155 (54.39%) respondents from the urban sector wanted to pursue post-graduation in OMFS. A total of 105 (36.84%) interns were interested in OMFS, compared to 85 (29.83%) third-year BDS students (Table 4).

**Table 4:** Participants' distribution according to reason for opting for OMFS as postgraduation (PG) course BDS: Bachelor of Dental Surgery; OMFS: Oral and maxillofacial Surgery; Data represented in form of n (%)

Reason to choose OMFS	n (%)	Personal choice, n (%)	Mentor influence, n (%)	Financial stability, n (%)	Career prospects, n (%)
Total responses	285 (63)	120 (42.10)	170 (59.65)	134 (47.01)	214 (75.08)
Type of institution					
Private	247 (86.67)	105 (36.84)	145 (50.88)	125 (43.86)	190 (66.67)
Government	38 (13.33)	15 (5.26)	25 (8.77)	9 (3.16)	24 (8.42)
Location of institution					
Rural	130 (45.61)	55 (19.30)	75 (26.32)	76 (26.67)	100 (35.09)
Urban	155 (54.39)	65 (22.81)	95 (33.33)	58 (20.35)	114 (40.00)
Level of study					
Intern	105 (36.84)	30 (10.53)	50 (17.54)	74 (25.96)	76 (26.67)
4th BDS	95 (33.33)	49 (17.19)	62 (21.75)	36 (12.63)	76 (26.67)
3rd BDS	85 (29.83)	41 (14.39)	58 (20.35)	24 (8.42)	62 (21.75)
Gender					
Male	95 (33.33)	35 (12.28)	35 (12.28)	65 (22.81)	72 (25.26)
Female	190 (66.67)	85 (29.82)	135 (47.37)	69 (24.21)	142 (49.82)

The mean KAP scores were significantly higher for government institutions in urban areas than for private institutions in rural areas (p≤ 0.05). The mean KAP scores were highest for interns, followed by final-year and third-year BDS students, with a statistically significant difference (p=.001). Males showed higher KAP scores than females; however, the difference was not statistically significant (p≥ .05), as shown in Table 5.

**Table 5:** Comparison of Knowledge, Attitude and Perception (KAP) scores in participants who chose OMFS (N=285). *p value ≤ 0.05: Significant using two-way Independent T Test; ** p value ≤ 0.05: Significant using two-way ANOVA test; CI: Confidence Interval; OMFS: Oral and Maxillofacial Surgery; Data presented in form of Mean ± Standard Deviation (SD)

Variables	N (%)	Knowledge score (Mean ± SD)	p-value	CI at 95% (upper limit, lower limit)	Attitude and perception score (Mean ± SD)	p value	CI at 95% (upper limit, lower limit)
Type of institution							
Private	247 (87.67)	3.67±1.21	0.011*	(-0.95, -0.12)	3.56±0.95	0.017*	(-0.72, -0.07)
Government	38 (13.33)	4.21±1.31			3.96±1.02		
Location of institution							
Rural	130 (45.61)	3.12±1.21	0.001*	(-1.03, -0.5)	3.56±1.03	0.002*	(-0.64, -0.14)
Urban	155 (54.39	3.89±1.08			3.95±1.12		
Level of study							
Intern	105 (36.84)	3.85±0.98	0.001**	(-1.74, -0.31)	3.65±1.08	0.001**	(-1.28, -0.02)
4th BDS	95 (33.33)	3.12±0.95			3.24±1.12		
3rd BDS	85 (29.82)	2.45±1.12			2.76±1.25		
Gender							
Male	95 (33.33)	4.12±1.45	0.12	(-0.10, 0.84)	4.34±1.96	0.11	(-0.09, 0.81)
Female	190 (66.67)	3.75±2.12			3.98±1.75		

## Discussion

Among the different branches of dentistry, OMFS stands out because of its dual qualifications and role as a bridge between medicine and dentistry. Contrary to a prevalent misconception, the responsibilities of an oral and maxillofacial surgeon extend beyond the treatment of the teeth and their immediate area. Their scope encompasses interventions aimed at improving the overall quality of life through the enhancement of form, function, and aesthetics, in addition to potentially life-saving interventions [9].

This survey focused on the awareness of undergraduate students opting for post-graduation in OMFS. A greater number of females participated in the survey than males. Previous studies have shown that women are increasingly drawn to dentistry, often due to their flexible schedule, which helps balance personal and professional lives [4,10,11]. This gender imbalance complicates the assessment of the true association between gender and interest in postgraduate studies.

Of these, 63% showed interest in pursuing postgraduate studies in OMFS, which is encouraging. This finding is in agreement with previous studies [10,12,13]. The early familiarity of dental students in India with both theoretical and clinical OMFS from their third year of training is responsible for their strong understanding of the scope of OMFS. This study indicated that early exposure to OMFS during undergraduate studies positively influences specialization choices, emphasizing the importance of early education in career decision-making. The main reasons for opting for postgraduation in OMFS were better career prospects and the influence of mentors. This might be due to the fact that as a specialized oral surgeon, one may find employment in various settings including hospitals, private clinics, or academic environments, typically receiving higher salaries in comparison to general dentists. Proficiency in conducting intricate procedures, such as maxillofacial surgeries, dental implants, and reconstructive surgeries, can distinguish individuals within the dental profession. Focusing on oral surgery can attract specific clientele seeking advanced medical attention. Skilled oral surgeons are often in demand globally, presenting opportunities for international work or involvement in humanitarian endeavors [12].

The results of this survey indicated that the clinical exposure during BDS and institutional exposure or guidance regarding their postgraduation created better awareness, along with counselling and mentoring being undertaken at the institutional level for students to help them make informed decisions about their future. Most respondents felt that OMFS has a great future and is more competitive than other specializations. This finding was supported by previous studies [4,5,10,12]. The main benefits of this branch were high earnings, reputation, and advanced skills, which were supported by the results of a study by Shah et al. [14].

It was further noticed that respondents from government institutions and urban areas had higher KAP scores. This could be due to the fact that government institutions and urban universities frequently possess superior research resources, which motivate students to participate in scholarly and clinical research. Urban areas and government institutions often enjoy enhanced access to state-of-the-art medical facilities and a substantial influx of patients, which are essential for the training of future oral surgeons [12,15]. More male interns showed higher KAP scores, which could be due to the increased exposure of interns to the various treatment modalities of OMFS during internship.

Our survey revealed that most female respondents were not willing to pursue post-graduation in OMFS. This finding was in agreement with previous studies [11,16]. According to these studies, sexism, marriage, family responsibilities, children, and society were the major career obstacles for females to practice and become oral and maxillofacial surgeons. According to the present survey, long working hours and associated risks and liabilities were the two main reasons for not opting for OMFS, which was also observed in previous studies [9,10,17].

OMFS training pathways vary globally and aim to meet the complex surgical demands of the specialty. Many countries have mandated dual qualifications or extended training programs that integrate medical teaching. In India, some centres practice the full scope of OMFS because of interactions with foreign experts, access to advanced equipment, and the efforts of individual surgeons. However, the current postgraduate curriculum prescribed by the Dental Council of India (DCI) does not fully align with modern OMFS requirements [15].

Limitations

The findings of our survey should be approached with caution because the majority of participants were primarily from a limited number of dental institutions in India, which introduces bias in the selection process. Additionally, the voluntary nature of participation may have resulted in a higher representation of students who were interested in career planning and surgery. Moreover, it is important to note that OMFS lacks a standardized training protocol on an international scale.

## Conclusions

In conclusion, the survey highlighted a significant interest among dental students specializing in OMFS, which underscores the unique appeal of this branch of dentistry as a discipline that bridges the gap between medicine and dentistry. The undecided status of many students also indicates the need for further guidance and information to help them make informed decisions about their future specialization. The mean KAP scores were higher for government institutions in urban areas and for males. Long working hours and associated risks and liabilities were the two main reasons for not opting for OMFS, whereas better career prospects, financial stability, and mentor influence were the main reasons for opting for postgraduation in OMFS.
